# Dr. James E. Crisfield

**Published:** 1905-03

**Authors:** 


Dr. James E. Crisfield, of Dansville, N. Y., a graduate of the College of Physicians and Surgeons, (N. Y.), 1873, died at Jacksonville, Fla., February 21, 1905, whither he had gone in the interest of his health. Dr. Crisfield was a prominent physician in the Genesee Valley and was reputed the oldest practising physician in Livingston county.

